# Functional Characterization of *Pneumocystis carinii* Inositol Transporter 1

**DOI:** 10.1128/mBio.01851-16

**Published:** 2016-12-13

**Authors:** Melanie T. Cushion, Margaret S. Collins, Thomas Sesterhenn, Aleksey Porollo, Anish Kizhakkekkara Vadukoot, Edward J. Merino

**Affiliations:** a University of Cincinnati College of Medicine, Cincinnati, Ohio, USA; b Cincinnati VAMC, Cincinnati, Ohio, USA; c Cincinnati Children’s Hospital Medical Center, Cincinnati, Ohio, USA; d Department of Chemistry, University of Cincinnati, Cincinnati, Ohio, USA

## Abstract

Fungi in the genus *Pneumocystis* live in the lungs of mammals, where they can cause a fatal pneumonia (PCP [*Pneumocystis* pneumonia]) in hosts with compromised immune systems. The absence of a continuous *in vitro* culture system for any species of *Pneumocystis* has led to limited understanding of these fungi, especially for the discovery of new therapies. We recently reported that *Pneumocystis carinii*, *Pneumocystis murina*, and most significantly, *Pneumocystis jirovecii* lack both enzymes necessary for *myo-*inositol biosynthesis but contain genes with homologies to fungal *myo*-inositol transporters. Since *myo*-inositol is essential for eukaryotic viability, the primary transporter, ITR1, was functionally and structurally characterized in *P. carinii*. The predicted structure of *P. carinii* ITR1 (PcITR1) contained 12 transmembrane alpha-helices with intracellular C and N termini, consistent with other inositol transporters. The apparent *K*_*m*_ was 0.94 ± 0.08 (mean ± standard deviation), suggesting that *myo-*inositol transport in *P. carinii* is likely through a low-affinity, highly selective transport system, as no other sugars or inositol stereoisomers were significant competitive inhibitors. Glucose transport was shown to use a different transport system. The *myo*-inositol transport was distinct from mammalian transporters, as it was not sodium dependent and was cytochalasin B resistant. Inositol transport in these fungi offers an attractive new drug target because of the reliance of the fungi on its transport, clear differences between the mammalian and fungal transporters, and the ability of the host to both synthesize and transport this critical nutrient, predicting low toxicity of potential inhibitors to the fungal transporter.

## IMPORTANCE

*myo*-Inositol is a sugarlike nutrient that is essential for life in most organisms. Humans and microbes alike can obtain it by making it, which involves only 2 enzymes, by taking it from the environment by a transport process, or by recycling it from other cellular constituents. Inspection of the genomes of the pathogenic fungi of the genus *Pneumocystis* showed that these pneumonia-causing parasites could not make *myo*-inositol, as they lacked the 2 enzymes. Instead, we found evidence of inositol transporters, which would import the sugar from the lungs where the fungi reside. In the present report, we characterized the transport of *myo*-inositol in the fungus and found that the transporter was highly selective for *myo*-inositol and did not transport any other molecules. The transport was distinct from that in mammalian cells, and since mammals can both make and transport *myo*-inositol, while *Pneumocystis* fungi must transport it, this process offers a potential new drug target.

## INTRODUCTION

Fungi belonging to the genus *Pneumocystis* live in the lungs of most mammals, where they can cause a fatal pneumonia in hosts with compromised immune systems (1). *Pneumocystis jirovecii* is the species that causes a lethal pneumonia (PCP [*Pneumocystis* pneumonia]) in humans, especially those with primary or acquired immune deficiencies (2). PCP continues to be a problem worldwide, even in the context of combined antiretroviral therapy in patients infected with HIV (3). Unexpectedly, the mortality rate of PCP after highly active antiretroviral therapy (HAART) remains unchanged, at about 15%, in HIV-positive populations in developed countries like the United States and England (2). However, the mortality rates in the developing world and certain urban areas of the United States underserved by the health care establishment approach 80% (4). Recent evidence correlating *P. jirovecii* as a comorbidity agent in respiratory conditions like chronic obstructive pulmonary disease (COPD) (5) or pneumonia in association with anti-tumor necrosis factor (TNF) antibody therapies (6) or with lung cancers (7) indicates a broadening of the population susceptible to PCP. In addition, patients with chronic inflammatory conditions are also showing increased infections due to concomitant corticosteroid therapy and other immunomodulators (8). Patients in these non-HIV-positive categories fare worse than those with HIV (9–11). The mainstay of PCP treatment and prophylaxis has long been trimethoprim-sulfamethoxazole (TMP-SMX). This treatment regimen can lead to severe allergic reactions (12), while *P. jirovecii* resistance (3) is being increasingly observed in the clinical management of these infections. Importantly, the lack of new therapies with which to treat colonized patients or those with PCP is a critical shortcoming.

It is well appreciated that the absence of a continuous *in vitro* culture system for any species of *Pneumocystis* has led to limited understanding of the life cycles, transmission, and natural histories of *Pneumocystis* fungi. These shortcomings have in turn led to a dearth of new therapies for treatment and prophylaxis of PCP. Since *Pneumocystis* fungi, and in particular *P. jirovecii*, lack ergosterol as the primary sterol component, the standard antifungal agents, such as the azoles and amphotericin B, are ineffective (13). The echinocandins, which target β-1,3-d-glucan synthesis, inhibit the formation of asci (cysts) but spare the more numerous proliferative life cycle stage, the trophic form (14). In previous work, we showed that after withdrawal of anidulafungin therapy, asci returned to the levels seen in untreated, *Pneumocystis murina*-infected mice, rendering this line of drugs ineffective as monotherapies (15). Thus, new targets are critically needed to treat this fungal pneumonia.

Recent advances in high-throughput sequencing techniques have facilitated investigation of the *Pneumocystis* and other fastidious microbes. We recently used comparative genomics to reveal that *Pneumocystis carinii*, *P. murina*, and most significantly, *P. jirovecii* lack both of the enzymes necessary for *myo-*inositol biosynthesis, inositol-3-phosphate synthase and inositol monophosphatase, which use glucose-6-phosphate as the initial substrate (16). The lack of these two enzymes was later upheld by another comparative genomics study of the three *Pneumocystis* species (17). This discovery is significant, as *myo-*inositol is a cyclic polyol that is critical for the pathogen’s survival. Thus, these fungi are inositol auxotrophs, and it follows that they must rely on sequestration of the host’s inositol. During scanning of the three *Pneumocystis* genomes, we identified sequences in all three that had homology to fungal inositol transporters (ITRs) (16). In *P. carinii* and *P. murina*, the species that infect rodents, two inositol transporter homologs (ITR1 and ITR2) were found, but the genome of *P. jirovecii* contained only a single such transporter, ITR1. We went on to show that both of the rodent ITRs were not only expressed during PCP but upregulated in comparison to the expression of other single-copy genes (16). Moreover, several other genes involved in inositol metabolism were also upregulated, indicating the importance of these pathways (16).

In the present study, we explored the predicted structure of *P. carinii* ITR1 (PcITR1), characterized the substrate specificity, pharmacology, and ion specificity of *myo-*inositol transport in *P. carinii*, and compared these to *myo-*inositol transport function in other fungi, protozoan parasites, and mammals. *P. carinii* organisms were used to characterize *myo*-inositol transport, as they are readily available from immunosuppressed rats. *P. jirovecii* is available in only limited numbers, and such studies could not be conducted using it at present.

We report that *myo-*inositol in *P. carinii* is likely obtained through a low-affinity, highly selective transport system. The transport is sufficiently distinct from mammalian transport systems that inhibitors which interrupt transport in these fungal pathogens will not likely affect transport in the host. In addition, since mammals and humans can synthesize *myo-*inositol, potential toxicity would be further reduced. Since *Pneumocystis* fungi can only obtain this essential nutrient through transport and the fungus infecting humans has but a single transporter to perform this function, *myo-*inositol transport presents an ideal new drug target.

## RESULTS

### Structure annotation of the *Pneumocystis carinii* inositol transporter.

**FIG 1:**
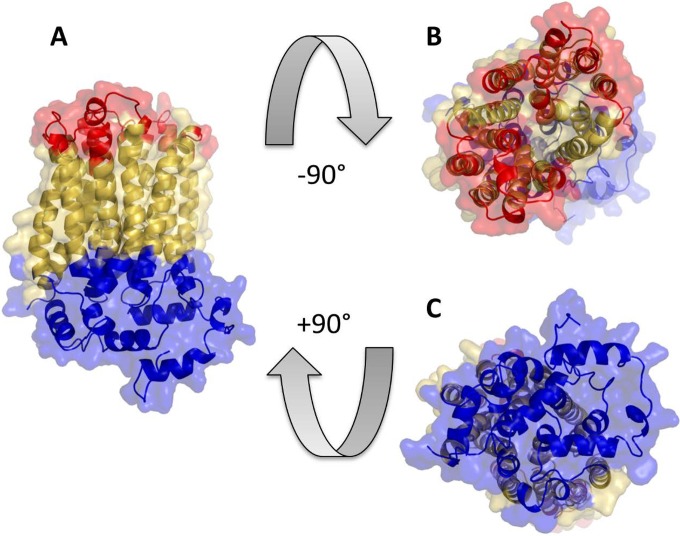
A 3-D model of PcITR1 in three projections. Colored yellow are residues predicted to be within the membrane-spanning regions. Residues predicted to be facing extracellular space are colored red. Residues colored blue are predicted to be cytosolic (for details on predictions, refer to Fig. S2 in the supplemental material).

At the time of manuscript preparation, Protein Databank did not contain resolved structures of any inositol transporters. Phyre2, with multiple templates, was therefore used to model the structures of the selected ITR1s. The resulting PcITR1 model (Fig. 1) has 93% of the residues modeled with over 90% confidence and is based on six combined templates (PDB identifiers [IDs] 4ybq_B, 5c65_A, 4gbz_A, 4pyp_A, 4lds_B, and 3j20_T; A, B, and T designate protein chains; the latter template was primarily used to model the C terminus only). The model indicates the presence of 12 transmembrane alpha-helical regions, forming a transport channel that is capped by nonmembrane-spanning alpha-helices (Fig. 1, blue) from one side, suggesting their possible role in regulating the permeability of the transporter. This suggestion is in line with the publications detailing the resolved structures used as templates to model PcITR1. GLUT5 (PDB ID 4ybq) and GLUT1 (PDB ID 4pyp) were proposed to have a gated-pore-type transport mechanism, where the intracellular helix bundle may work as a latch to keep the transporter gate in the outward-facing conformation (18, 19). Comparison with 3-dimensional (3-D) models of other selected ITRs (listed in Materials and Methods) supports the topology of the inositol transporter with 12 membrane regions. All models have the transmembrane regions well aligned, with root mean square deviation (RMSD) values ranging from 2.6 to 3.3 Å, showing differences only in their termini beyond the membrane domain (see Fig. S1 in the supplemental material).

A number of sequence-based and one structure/sequence consensus-based prediction methods were employed to evaluate the membrane topology of PcITR1 and to determine cytosolic and extracellular parts of the membrane protein. All methods agree on the 12-alpha-helix topology of the transporter, and most indicate that both N and C termini are located in the cytoplasm (see Fig. S2 in the supplemental material). The NCBI Conserved Domain Database (CDD) indicates that the sequence has the highest homology to the following domains: sugar (and other) transporter (E value = 1.53 × 10^−103^), major facilitator superfamily (MSF) transporter (E value = 3.36 × 10^−93^), and d-xylose transporter (E value = 2.94 × 10^−60^). The other selected inositol transporters share only 25 to 27% sequence identity with PcITR1. Nevertheless, CDD shows that they all yield top homology to the same conserved domains as does PcITR1.

### The *K_m_*^app^ of *P. carinii myo*-inositol uptake

**FIG 2:**
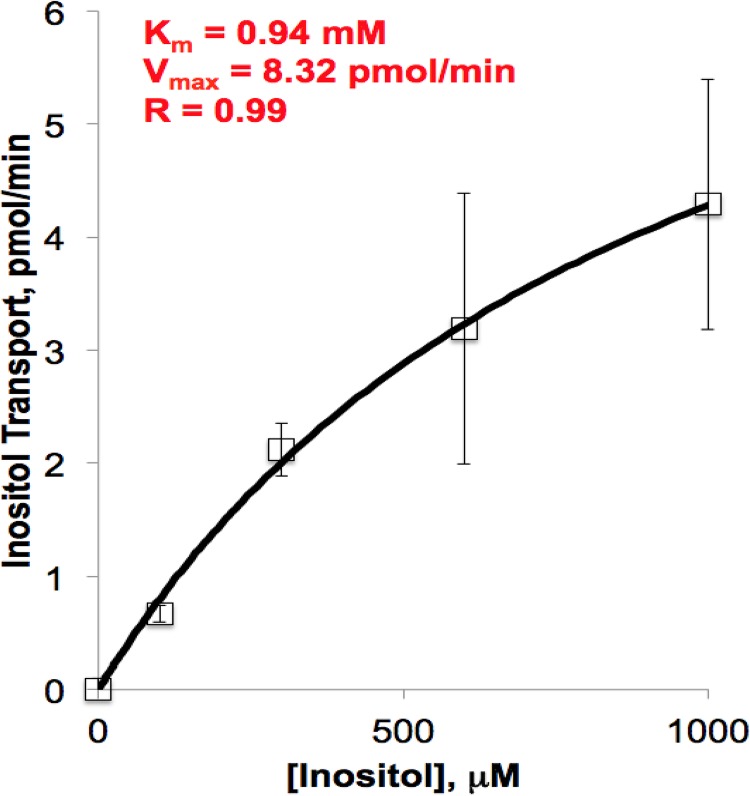
Substrate saturation kinetics of *myo*-inositol uptake in *P. carinii*. Uptake of *myo*-[2-^3^H]inositol in *P. carinii* cells was monitored at concentrations of between 50 µM and 1.5 mM *myo*-inositol at pH 7.0 in PBS. Nonlinear regression was used to calculate an apparent *K*_*m*_ of 0.94 mM, *V*_max_ of 8.32 pmol/min, and *R* of 0.99. Error bars show standard deviations.

*myo-*Inositol uptake by *P. carinii* was time dependent and saturable. The apparent Michaelis constant (*K_m_*^app^) was calculated from the linear uptake of *myo-*inositol by *P. carinii* at varying concentrations and at differing time points, following a modification of the methods of Jin and Seyfang (20). Nonlinear regression analysis was used on the averaged results of six biological replicates. The inositol concentrations ranged from 10 μM to 1 mM to mimic the physiological range and avoid the higher concentrations where passive diffusion is possible. The *K*_m_^app^ was found to be 0.94 ± 0.08 mM (mean ± standard deviation), with a *V*_max_ of 8.3 ± 0.5 pmol min^−1^ per 5 × 10^7^ *P. carinii* cells (Fig. 2). The *K_m_*^app^ is within four- to twofold those of *myo-*inositol transport in other fungi. *Candida albicans* has a reported *K*_*m*_ of 240 ± 15 µM (20), *Schizosaccharomyces pombe* a *K*_*m*_ of 260 ± 4 µM (21), the protozoan parasite *Leishmania donovani* a *K*_*m*_ of 250 ± 5 µM (22), and *Saccharomyces cerevisiae* a reported *K*_*m*_ range of 100 to 470 µM (23). Linearization of the data by Hanes plot transformation revealed a relatively good fit with an *R* value of 0.92 within the substrate range tested (data not shown), demonstrating that *myo-*inositol uptake in *P. carinii* occurs by a single or dominating high-affinity transport system, since a value of 1 would indicate the absence of cooperativity. Thus, ITR1 is the major protein responsible for inositol uptake and has slightly weaker affinity than other inositol transporters. To further investigate this transporter, we decided to investigate its selectivity.

### *myo-*Inositol transport in *P. carinii* is proton dependent and sodium independent.

**FIG 3:**
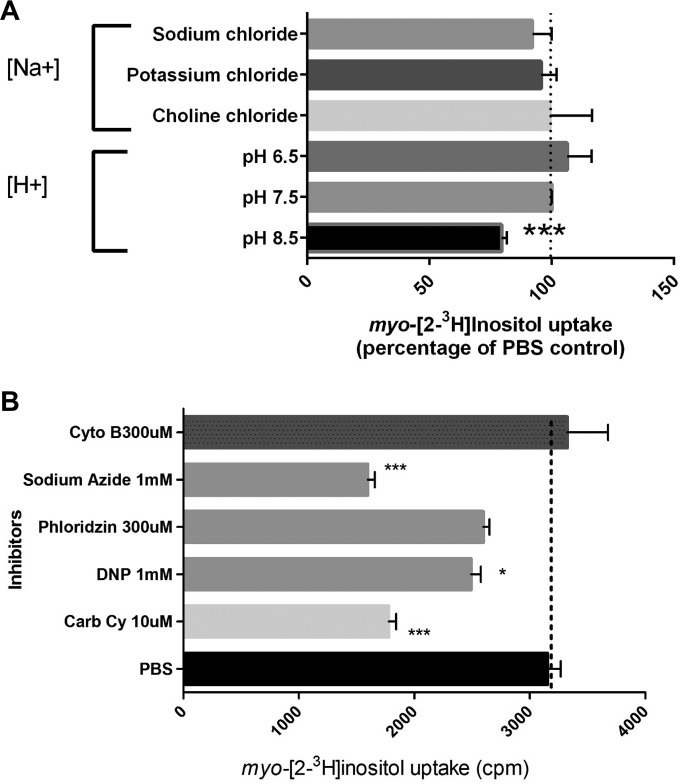
Ion specificity and pharmacology of *myo-*inositol transport in *P. carinii*. (A) Ion specificity. *myo*-[2-^3^H]inositol uptake was measured in the presence of different ion buffers and at pHs ranging from acidic to basic. Data are expressed as the percentage of the result for the PBS control. Significance was determined by one-way analysis of variance (ANOVA). ***, *P* < 0.001. (B) Pharmacology. *myo*-[2-^3^H]inositol uptake was measured in the absence (PBS control) or presence of various inhibitors. Data are expressed as counts per minute (cpm). Significance was determined by one-way ANOVA. *, *P* < 0.05; ***, *P* < 0.001. Cyto B, cytochalasin B; DNP, dinitrophenol; Carb Cy, carbonilcyanide; PBS, phosphate-buffered saline. Error bars show standard deviations.

As most drugs used in humans for control of diabetes target the sodium-dependent transporters, it is important to characterize the ion specificity of the *myo-*inositol transport in *Pneumocystis* fungi to better target potential inhibitors (24). *myo*-Inositol uptake was evaluated in the presence of different proton concentrations (pH) and other inhibitors to identify the ion specificity of its transporter, using uptake of *myo*-[2-^3^H]inositol as the readout (Fig. 3A). The sodium independence of *P. carinii myo-*inositol transport was clearly demonstrated by uptake in a sodium-free buffer and replacement of NaCl by KCl or choline chloride without a difference in transport capability. In contrast, *myo-*inositol transport was shown to be pH dependent, as illustrated by a significant difference at pH 8.5, where the H^+^ proton concentration is 3.2 nM, compared to 316 nM at pH 6.5. This further differentiates the *P. carinii myo-*inositol transport from the sodium-dependent *myo-*inositol transporters (SMITs) in mammalian cells and shows that it has the same characteristics as the high-affinity *myo-*inositol transporter in *C. albicans* (20).

### Pharmacology of *myo-*inositol transport in *P. carinii* differs from that of *myo-*inositol transport in mammals.

*P. carinii* uptake of radiolabeled *myo-*inositol was measured in the absence or presence of the protonophores carbonilcyanide and dinitrophenol, which are thought to exert their effects by collapsing membrane proton gradients. Both exhibited a significant effect on *myo-*inositol transport in *P. carinii* (43.5 and 21%, respectively), strongly suggesting that the transport is proton coupled (Fig. 3B). The inhibition of transport was pronounced with sodium azide (~50%), indicating that the process is energy dependent, as azide reduces ATP levels by disruption of ion pumps that maintain a electrochemical proton gradient across membranes. Cytochalasin B and phloridzin (also called phlorizin), which act by inhibiting transporters that operate by facilitated diffusion, like the mammalian SGLT1 and SGLT2, had no or little effect on uptake (5.4 and 18% increase, respectively), suggesting that this mechanism of transport does not affect *myo-*inositol transport. Phloridzin is the more potent glycoside of phloretin, which has been found to inhibit GLUT2 and a variety of urea transporters.

### *P. carinii* ITR1 is highly specific at physiological concentrations of competing substrates.

Substrate specificity is a critical defining attribute that characterizes different transporters, and its determination is necessary for future drug design efforts. Biochemical characterization was also critical to perform here, as the specificity of these transporters in *Pneumocystis* fungi is in question (17). The substrate specificity of *P. carinii myo-*inositol transport was determined by testing 8 stereoisomers, including *myo-*inositol, 4 inositol derivatives, and 14 sugars for their ability to compete with *myo-*inositol uptake. Stereoisomer assays were performed with 5 μM radiolabeled inositol and 500 μM competing substrate; sugars were added at 10 mM.

### Stereoisomer specificity.

**FIG 4:**
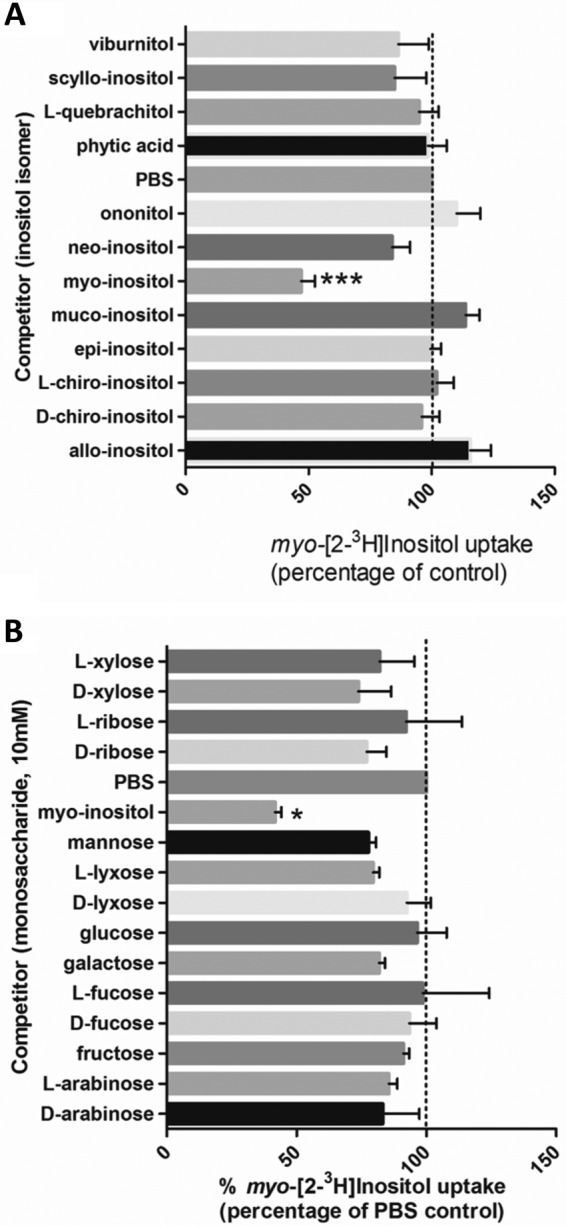
Isomer and sugar specificity of *myo-*inositol transport. (A) Isomer specificity. Competition for uptake of *myo-*inositol and 5 inositol derivatives was tested. Concentrations of 0.5 µM *myo*-[2-^3^H]inositol and 100× the unlabeled inositol isomers were used. Data are the average results from 5 separate analyses. Data are expressed as percentages of the results for the PBS control (no competitor). Significance was determined by the paired-sample two-tailed *t* test. ***, *P* < 0.001. (B) Sugar specificity. Competition for uptake of *myo-*inositol and 14 common sugars was tested. Concentrations of 0.5 µM *myo*-[2-^3^H]inositol and 100× the unlabeled inositol isomers and sugars were used. Significance was determined by the paired-sample *t* test with two-tailed *P* values. *, *P* < 0.05; ***, *P* < 0.001. Error bars show standard deviations.

To define the substrate specificity of *P. carinii myo-*inositol transport, we explored the abilities of the eight stereoisomers of *myo-*inositol, as well as 4 inositol derivatives, to compete with *myo*-[2-^3^H]inositol in uptake in the absence or presence of a 100-fold excess of the unlabeled isomers (Fig. 4A). *myo-*Inositol is a polyol in which each of the six carbons is hydroxylated. The stereoisomers of *myo-*inositol differ in the configuration of their hydroxyl groups above (*cis*) or below (*trans*) the carbon ring, with *myo-*inositol as *cis*-1,2,3,5-*trans*-4,6-cyclohexanehexol, *scyllo-*inositol as 1,3,5/2,4,6-hexahydroxycyclohexane, *neo*-inositol as 1,2,3/4,5,6-cyclohexanehexol, *muco*-inositol as 1,2,4,5/3,6-cyclohexanehexol, d-*chiro*-inositol as 1,2,5/3,4,6 hexahydroxycyclohexane, l-*chiro*-inositol as 1,4,5/2,3,6 hexahydroxycyclohexane, *epi*-inositol as 1,2,3,4,5/6-hexahydroxycyclohexane, and *allo*-inositol as 1,2,3,4/5,6 hexahydroxycyclohexane.

Our studies found that the only isomer able to compete for transport with radioactive *myo-*inositol uptake by *P. carinii* was the unlabeled *myo-*inositol. *neo-*Inositol and *scyllo*-inositol showed some inhibition, though none of the results reached statistical significance, indicating that even slight changes in stereochemistry are not well tolerated. In addition, none of the derivatives, including viburnitol (1-d-3-deoxy-*myo-*inositol), l-quebrachitol (2-*O*-methyl-l-*chiro*-inositol), phytic acid (*myo-*inositol-1,2,3,4,5,6-hexakisphosphate), and d-pinitol (3-*O*-methyl-d-*chiro*-inositol) (data not shown), were able to significantly inhibit the uptake. This striking selectivity under physiological conditions is very similar to that of the *Trypanosoma brucei* HMIT, which tolerates only a single modification on the inositol ring by elimination of the hydroxyl group at the C-1 or C-6 position, as determined by electrophysiological assessment (25). Inversion of the hydroxyl groups was partially tolerated, as evidenced by some activity remaining with *scyllo*-, *epi*-, l-*chiro*-, and d-*chiro*-inositol in the same study. However, an inversion with a substitution (deoxy or methyl ether) suppressed all activity, as was observed with quebrachitol. The only stereoisomer recognized by the *C. albicans myo-*inositol transport system was *neo*-inositol, suggesting that the hydroxyl at carbon 5 is not important for recognition of substrate. No inhibition was observed for *scyllo*- or d-*chiro*-inositol, which differ from *myo-*inositol only by the hydroxyl at carbon 2 or 3, respectively, suggesting that both of these hydroxyls are critical for recognition and binding (20). Likewise, the hydroxyl in *trans* at carbon 4 in the *C. albicans* system appears to be important for recognition and binding, since weak or no inhibition was observed with *epi*-inositol or *allo*-inositol. Based on the studies presented here, no changes in stereoisomer structure or substitution would allow competition with the radiolabeled *myo-*inositol, suggesting very high affinity of *myo-*inositol transport in *P. carinii*.

### Sugar substrate specificity.

When evaluating *myo-*inositol transport, it is necessary to consider monosaccharides, hexoses, and pentose sugars because they are also present in high concentrations in the host. Sugar substrate selectivity was probed in the same manner described for stereoisomers and in the presence of 10 mM of unlabeled sugars as potential competitors (Fig. 4B). The four hexose sugars tested were d-glucose, d-mannose, d-galactose, and d-fructose. Slightly decreased effects on uptake were observed with the latter 3 sugars, but these did not reach significance. The pentose sugar d-xylose is structurally closest to *myo-*inositol and potentially the closest competitor, but it had no significant effect on uptake, nor did its isomer or the other pentose sugar isomers ribose, lyxose, and arabinose (all aldopentoses) or the deoxy-aldohexoses, d- and l-fucose. In contrast, the addition of unlabeled *myo-*inositol significantly inhibited the uptake, by ~60%, attesting to the specificity of this transporter. The stereoisomer and sugar findings are congruent in their identification of the high substrate specificity of *myo-*inositol transport in *P. carinii* under these test conditions. It is notable that the pyranose l-fucose or its stereoisomer d-fucose did not have any effect, because l-fucose is increased in diabetic patients and is a strong competitor and substrate for the human SMIT. l-fucose and d-xylose are inhibitors of the Na^+^-dependent inositol transport system in humans. Thus, the Na^+^ independence of *myo-*inositol transport (Fig. 3A) and the lack of effect of l-fucose on the viability of *P. carinii* (Table 1) underscore the fact that the *P. carinii myo-*inositol transport system is highly selective and Na^+^ independent.

**TABLE 1:** *In vitro* assessment of potential transport inhibitors

Compound tested, concn (µg/ml)	% reduction in ATP vs. untreated control after 72 h
Lithium chloride	
500	0
250	0
125	0
62.5	0
l-Fucose	
1,000	0
100	0
10	0
1	2.55
0.1	3.37
Valproate	
100	11.68
10	1.91
1	20.62
0.1	13.73
Amitriptyline	
100	99.61
10	98.76
1	4.37
0.1	0
Carbamazepine	
100	69.30
10	6.41
1	0.07
0.1	0

**FIG 5:**
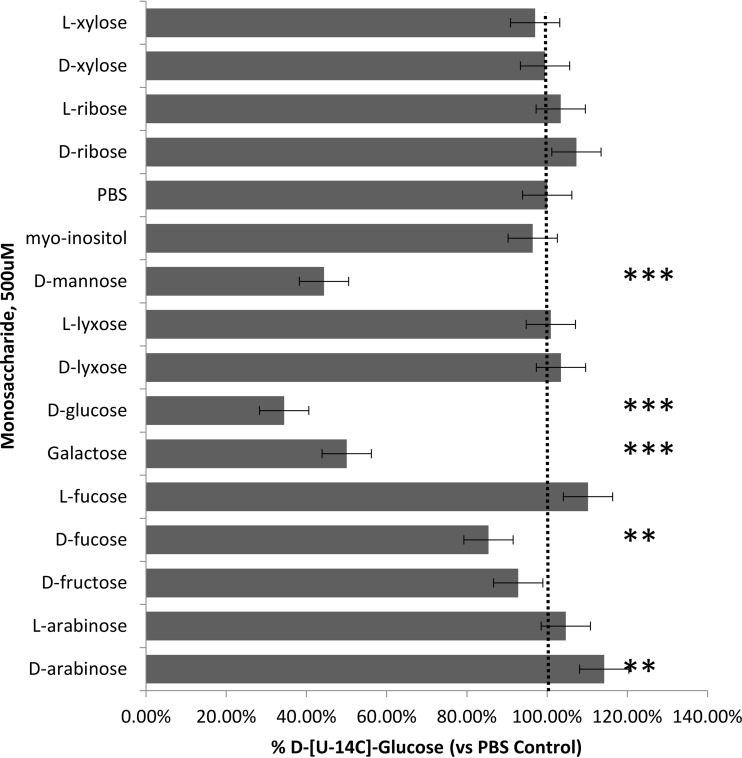
d-[U-^14^C]glucose uptake by *P. carinii*. Competition for uptake of *myo-*inositol and 14 common sugars was tested. Concentrations of 5 µM D-[U-^14^C]glucose and 100× the unlabeled inositol isomers and sugars were used. Significance was determined by the paired-sample *t* test with two-tailed *P* values. Error bars show standard deviations.

To further establish the specificity of *myo-*inositol transport in *P. carinii*, the uptake of ^14^C-d-glucose was evaluated in the context of monosaccharide inhibitors in much the same manner used for assessing the specificity of *myo-*inositol uptake. As shown by the results in Fig. 5, transport of the radiolabeled glucose was not inhibited by the addition of unlabeled *myo-*inositol, but the hexose sugars glucose (~70%), mannose (~50%), and galactose (~50%), as well as d-fucose (15%), were all able to inhibit the uptake, Interestingly, D-arabinose significantly stimulated glucose uptake, by about 15%.

### Effects of putative inhibitors of *myo-*inositol synthesis and transport on viability of *P. carinii*.

**FIG 6:**
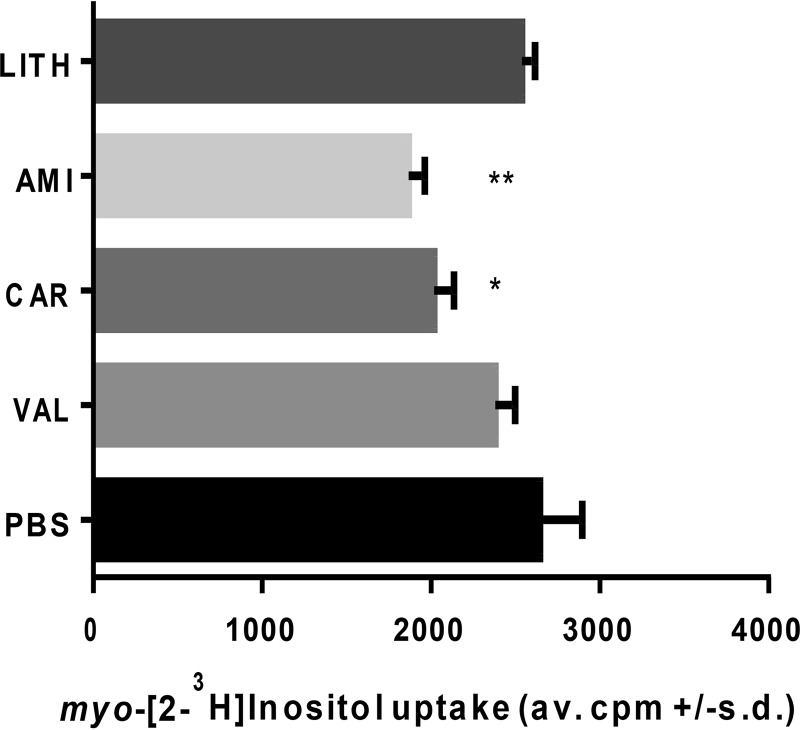
Effects of putative inhibitors of *myo*-inositol biosynthesis and transport. *P. carinii* was incubated with a concentration of 100 µg/ml of amitriptyline (AMI; 3.3 mM), carbamazepine (CAR; 4.25 mM), or valproate (VAL; 6.25 mM) or 500 µg/ml of lithium chloride (LITH; 117 mM) for 10 min prior to the addition of radiolabeled *myo*-inositol as described in Materials and Methods. Significance was determined by the paired-sample *t* test with two-tailed *P* values. Error bars show standard deviations.

Studies on the inhibition of *myo-*inositol transport and interruption of *myo-*inositol biosynthesis have explored them as potential therapies for bipolar disease. Standard treatments of this neurological disease include lithium salts, (e.g., lithium chloride), carbamazepine, and valproate (26). Studies have hypothesized both that lithium can inhibit *myo-*inositol biosynthesis and that it can decrease the activity of high-affinity *myo-*inositol transport, along with 2 other bipolar therapies, valproate and carbamazepine, based on studies in cultured astrocytes (26). We evaluated the effects of these drugs on the uptake of radiolabeled *myo-*inositol (Fig. 6) and on the viability of cultured *P. carinii* using an ATP-driven assay (Table 1) (27). As shown by the results in Fig. 6, lithium and valproate had no effect on uptake, while amitriptyline and carbamazepine reduced uptake by about 30% and 20%, respectively.

To evaluate whether these compounds had a correlative effect on the viability of *P. carinii*, an *in vitro* assessment using live organisms was then conducted. Table 1 shows the results of the inhibitors after 3 days of exposure to *P. carinii* in a cell-free system, with the readout of ATP measured by relative light units (RLU) in a luciferine-luciferase-based assay (28). Included in these assays was the sugar l-fucose, which has been shown to inhibit the human SMIT transporters. *P. carinii* was unaffected by exposure to lithium chloride at concentrations as high as 500 µg/ml, which would be consistent with the lack of synthetic activity in *P. carinii*. Likewise, valproate and l-fucose had no significant effects on ATP levels. In contrast, carbamazepine reduced the ATP content by 70% at the highest concentration, though lower levels did not exert any influence. Carbamazepine was shown to reduce radiolabeled *myo-*inositol transport in astrocyte cell cultures (29). Of interest was the effect of amitriptyline, a tricyclic antidepressant which acts primarily as a serotonin-norepinephrine reuptake inhibitor, with strong actions on the serotonin transporter and moderate effects on the norepinephrine transporter (29). Besides identifying potential new anti-PCP therapies, these studies showed the correlation between the 2 assay systems. Lithium and valproate were unable to block radiolabeled *myo-*inositol uptake (Fig. 6) and also had no effect on the viability of *P. carinii* in the ATP assay system (Table 1), while amitriptyline and carbamazepine did block uptake (Fig. 6) and were able to reduce viability as assessed by ATP levels (Table 1). However, the normal therapeutic target levels for humans are 120 to 150 ng/ml for amitriptyline and 4 to 12 µg/ml for carbamazepine. These levels are much lower than those used in these short-term experiments, and the inhibition observed could have been due to other, off-target effects. Nonetheless, there was some selectivity exhibited by these 2 drugs.

## DISCUSSION

Eukaryotic cells typically obtain *myo-*inositol from three sources: they can make it from glucose-6-phosphate using just 2 enzymes, 1-d-*myo-*inositol-phosphate synthase and inositol monophosphatase, they can sequester it from extracellular sources by specialized *myo-*inositol transporters, or it can be obtained by dephosphorylation or recycling of inositol-containing membrane or cellular phospholipids (30). *myo*-Inositol is involved in at least three essential cellular functions. As a constituent of phosphatidylinositol, it is a component of biological membranes, and when phosphorylated, it participates in exocytosis, cytoskeletal restructuring, and intracellular membrane trafficking; it is critical in signal transduction pathways that control many cellular functions; and lastly, it is a precursor of glycosylphosphatidylinositol (GPI) membrane anchors, which affix glycolipids to cell surfaces (31). If *Pneumocystis* cannot obtain its *myo-*inositol by *de novo* synthesis, we hypothesized that these fungi must transport it from its environment within the mammalian lung. In keeping with that supposition, we identified 2 potential inositol transporters in the genomes of *P. carinii* and *P. murina* and a single one in *P. jirovecii* (16).

### Characterization of *P. carinii myo*-inositol transport and comparisons to mammalian and fungal transport systems.

PcITR1, as well as other ITRs analyzed in this work, appears to belong to the major facilitator superfamily (MFS) of membrane proteins that are expressed ubiquitously across all phyla and are involved in the transport of various small-molecule compounds (32). The superfamily comprises at least 17 distinct families that generally show specificity to a single class of compounds, including simple sugars (hexoses, pentoses, and disaccharides), inositols, amino acids, nucleosides, various organic and inorganic cations and anions, and even some drugs. The MFS fold consists of two 6-transmembrane helix bundles that are connected by intracellular helices or extended loops. These two 6-helix bundles can adopt a clamshell-like outward opening with a hydrophilic cavity, where the amino acids lining this cavity determine the transporter’s specificity for a certain class of substrates (19, 33-35). Once a substrate is docked into the cavity, the MFS transporter changes the conformation of the 6-helix bundles to open the cavity inward and simultaneously close the outward cavity through a so-called “rocker switch” mechanism (also known as the alternating access mode of operation), which prevents a continuous permeability across the membrane (34, 35). In this work, we characterized the kinetics, substrate specificity, and possible inhibition of PcITR1 to help define the function of this essential system.

Next, we biochemically characterized *myo*-inositol transport in *P. carinii*. The apparent *K*_*m*_ of 0.94 mM suggests that it would be considered a low-affinity but highly selective transporter. Similar transporters in other fungi (discussed below) have somewhat stronger affinities. We propose two explanations for these finding. First, the PcITR1 may have evolved to be a selective binder rather than a strong binder. Thus, the lower *K*_*m*_ may allow stringent control of this essential nutrient, as it has no other source except that in the external environment. The need to control *myo*-inositol transport could occur as a result of increasing damage to the lung as the pneumonia progresses, resulting in denudation of the alveoli and thus permitting leakage of blood components, including *myo*-inositol, into these compartments. Reports of the concentrations of free *myo*-inositol in human serum vary widely, from 38 µM in adult females (36) to 823 µM in preterm infants provided inositol supplements (37), and the selectivity demonstrated by *P. carinii* ITR1 may reflect a physiological response to the changing *myo*-inositol environment. An example of a similar situation can be found in the human GLUT-2 glucose transporter, whose low *K*_*m*_ (15 to 20 mM) allows it to change its transport rate in proportion to the increased glucose concentrations that occur postprandially (38).

The second possibility is the role that alternative stages in the *P. carinii* life cycle may play in different uptake efficiencies. Since the organisms are isolated directly from the lungs of infected rats, all life cycle stages are present during the uptake experiments. There may be differential uptake abilities and/or kinetics for the different stages. A dormant stage, possibly the ascus, could detrimentally affect the total uptake, depending on the number present in the lung preparation, as we express our data as per 5 × 10^7^ nuclei, counting all stages in the sample. A single mature ascus contains 8 nuclei, though the trophic forms typically outnumber the asci by a factor of 10:1. Thus, if almost half of the nuclei are not transporting the substrate, this could skew the results.

Our data provide strong evidence that *myo-*inositol transport is a sodium-independent process in *P. carinii*, that it requires energy, and that it is proton dependent. Replacing sodium with potassium or choline did not affect transport; however, the reduction of protons at pH 8.5 significantly reduced transport. Moreover, the protonophores dinitrophenol and carbonilcyanide, which uncouple the proton gradient in the electron transport chain, significantly inhibited *myo-*inositol transport, as did sodium azide, signaling its energy dependence. Phloridzin did not significantly inhibit transport, again differentiating it from the mammalian sodium-dependent glucose transporters.

In humans, *myo-*inositol is taken up from the environment by sodium-dependent *myo-*inositol transporters 1 and 2 (SMIT1 and -2), which cotransport it with two sodium ions along the concentration gradient, and by the H^+^-dependent transporter HMIT, which cotransports it with H^+^ (24). SMIT1 (encoded by gene *SLC5A3*) and SMIT2 (SGLT6, alias *SLC5A11*) belong to a larger family of sodium glucose transporters, SGLT1 to -6, which are sodium-glucose symporters, encoded by *SLC5A1*, *A2*, *A4*, *A9* and *A10* genes, and generally are associated with transport of sugars in the digestive tract and kidneys. They are localized to the plasma membrane and can also transport xylose and glucose. SMIT1 and -2 are highly expressed in the human brain, where inositol levels are thought to be about 100-fold greater than in other parts of the body, at 2 to 25 mM (24, 39). The SGLT cotransporter family transport a number of sugars, including glucose, galactose, mannose, and fructose, as well as *myo-*inositol, via SGLT6/SMIT. SMIT1 transports l-fucose, l-xylose, and d- and l-glucose. SMIT2 transports d-chiro-inositol, d-glucose, and d-xylose. Both SMITs are phloridzin sensitive. Based on their sodium dependence, sugar specificities, and sensitivity to phloridzin, they are distinct from the *myo*-inositol transporters in *P. carinii*.

The second major family of transporters in mammals are the glucose transporters (GLUTs). There are 14 isoforms that share structural features, such as 12-transmembrane domains and intracellular-facing N and C termini (24). The proton-driven *myo-*inositol transporter HMIT (GLUT13) in humans belongs to the class III GLUT family, whose members are defined by their protein sequence homologies, substrate specificities, and tissue localization. GLUT13 is the only transporter of *myo-*inositol in this family. The differences between the human HMIT and the *P. carinii* transporter are many. HMIT is restricted to an intracellular location, while the ITR1 of *Pneumocystis* localizes to the periphery of the cells, presumably to the plasma membrane, as predicted by its sequence structure. HMIT has an apparent *K*_*m*_ of 100 µM and was inhibited by cytochalasin B and phloridzin (24), while the *P. carinii* transporter was not. These results indicate that inhibitors directed to the fungal *myo-*inositol transport should not interfere with mammalian transporters and that we have not inadvertently evaluated mammalian transporters in this study as potential contaminants in the fungal preparations.

Most fungi in which *myo-*inositol transport has been studied appear to be able to both synthesize *myo-*inositol and transport it via a single or multiple specific transporters. *Saccharomyces cerevisiae* can both synthesize *myo-*inositol and import it via the products of two distinct transporter genes, the high-affinity ITR1 and the lower-affinity ITR2, which are sodium independent and energy dependent, with reported *K*_*m*_s ranging from 0.1 mM to 0.47 mM (23). *C. albicans* also has the ability to synthesize *myo-*inositol but has only a single high-affinity inositol transporter (20). The *C. albicans* transporter (*K*_*m*_ of 0.240 mM) was also shown to be sodium independent, energy dependent, and insensitive to inhibitors of facilitated diffusion, like the transporters in *P. carinii*. Remarkably, *Cryptococcus  neoformans* possesses 10 to 11 inositol transporters, depending on the variety of the fungus (40), which is thought to result from the requirement for inositol from plants in its native environment. Two of these, ITR1A and ITR3C, are suggested to be the primary inositol transporters within this large family (41). These transporters were functionally characterized in yeast mutants (41) but remain to be analyzed for substrate specificity. *Schizosaccharomyces pombe*, which like *Pneumocystis* fungi, is a *myo-*inositol auxotroph, has 2 transporters, ITR1 and ITR2, with a *K*_*m*_ of 0.260 mM (20).

The *myo*-inositol transport in some protozoan parasites has been characterized. In *Trypanosoma cruzi*, the etiologic agent of Chagas disease or American trypanosomiasis, *myo-*inositol transport is facilitated by an energy-dependent, phloridzin-sensitive but cytochalasin B-insensitive system, suggesting that there are at least 2 transporters, a sodium-dependent and a sodium-independent one, that are operational in these parasites (42). *Leishmania donovani*, the agent of leishmaniasis, transports *myo-*inositol with a single transporter, *L. donovani* MIT (LdMIT), with a *K*_*m*_ of 0.08 mM (43). Like the fungal transporters, LdMIT is a *myo-*inositol proton symporter driven by a proton gradient and is expressed at the plasma membrane (22).

*C. albicans* has a number of opportunities to salvage inositol from its human host. As an intestinal commensal, it has access to the dietary intake of *myo-*inositol, which is about 1 g/day, making for a reliable source (20). In the disseminated state, it can be exposed to 15 to 70 µM in human serum and up to 200 to 270 µM in liver and lymphatic tissue. In infections with *C. albicans* and *C. neoformans*, *myo-*inositol is associated with pathogenesis and virulence (20). In the case of *C. neoformans*, *myo-*inositol secreted by plants stimulates mating, as does exogenous *myo-*inositol imported by transporters in *S. pombe* (21). In *Trypanosoma brucei*, the H^+^-linked *myo-*inositol transporter *T. brucei* HMIT (TbHMIT) was recently shown to be essential for viability in its bloodstream form (25). In *L. donovani*, a cytotoxic *myo-*inositol analogue was shown to dramatically reduce viability (44).

Within the lung, *Pneumocystis* is exposed to rich sources of *myo-*inositol in its free form (45) or as cleavage products of inositol-containing molecules, such as phosphatidylinositol or phospholipids (45). Thus, these fungi may have additional means to import inositol-containing molecules that could then be metabolized to release the inositol, entering into further metabolic pathways. The upregulation of genes associated with inositol metabolism described in our previous paper provides some support for this contention (16). The connection of inositol with mating in *S. pombe* and *C. neoformans* is additionally intriguing, as *Pneumocystis* species reproduce sexually within the lung (1). All of these studies attest to the essentiality of this simple sugar and its potential as a new target.

### *Pneumocystis myo-*inositol transport is highly selective.

In contrast to *myo-*inositol transport in *C. albicans*, *L. donovani*, and *T. brucei*, the *Pneumocystis myo-*inositol transport system was selective for *myo-*inositol only and did not transport other stereoisomers or sugars that were evaluated in several experiments. To test this specificity in another way, we conducted transport studies with radiolabeled d-glucose. Transport was significantly inhibited by d-mannose, d-glucose, d-galactose, and d-fucose but not *myo-*inositol, providing evidence that there is a hexoselike transporter that is separate and distinct from the transport of *myo*-inositol.

Inositol and the management of its levels in the human brain are a focus of investigation for the treatment of neurological disorders like bipolar disease, where it is hypothesized that reduction of cellular signaling via inhibition of *myo-*inositol biosynthesis or reduction of its cellular concentrations reduces or prevents symptoms (29, 43). Inositol monophosphatase is one putative target for lithium therapy, one of the primary treatments for bipolar disease. Valproate and other mood stabilizers, such as carbamazepine, have also been investigated for their mechanisms of action, which appear to act in shared and disparate cellular processes. SMIT1 activity was inhibited by lithium, valproate, and carbamazepine, but it is not likely to be the primary target of these mood stabilizers (29). We report here that the uptake of radiolabeled *myo-*inositol by *P. carinii* was unaffected by the addition of lithium salt, nor was lithium salt effective in reducing the viability of the fungus after 3 days of exposure, even at high levels. Such results would be expected if the target is biosynthesis of *myo-*inositol. Valproate was also ineffective at blocking transport and reducing viability. The inhibition of uptake and reduction of viability by amitriptyline and carbamazepine were unexpected, though both had modest effects in each assay and were present at levels exceeding target therapeutic doses. The mechanisms of action of both compounds are under active investigation, but a role for inhibition of *myo-*inositol transport has not been reported, and the effects on *P. carinii* may be due to a downstream or heretofore-unidentified mechanism. Nonetheless, further investigations of the effects of these compounds on *Pneumocystis* infection in an animal model of PCP seem warranted.

## MATERIALS AND METHODS

### Source of *Pneumocystis*.

Stocks of *P. carinii* were prepared from individual rats, assessed for microbial contamination, ATP content, and organism numbers, and then cryopreserved in liquid nitrogen, as we have described previously (46). For use in uptake studies, cryopreserved organisms were quickly thawed at 37°C, plated in RPMI 1640 with 20% horse serum and other nutritional supplements (47), and treated with antibiotics and antimycotics for 24 h to further reduce the possibility of microbial contamination.

Suppression of the immune system of rats was induced by intraperitoneal injections of methylprednisolone (0.01 to 0.02 mg/kg of body weight subcutaneously once per week) for up to 8 to 10 weeks. Water was acidified to prevent any secondary bacterial infections. Rats were used as a source of the organisms, as they produce higher organism burdens with larger *Pneumocystis* numbers than mice and fewer rats are needed to maintain stock levels. All procedures were conducted according to IACUC-approved protocols. *P. carinii* organisms were purified from lung tissue by a series of centrifugations and a lytic step to reduce erythrocytes and host lung cells (46).

The studies involving animals were conducted in compliance with applicable laws, regulations, and institutional policies and with approval by the institutional animal care and use committees of the University of Cincinnati and the Cincinnati Veterans Affairs Medical Center (M. T. Cushion holds the protocols).

### Reagents.

*myo*-[2-^3^H]inositol (specific activity of 21 to 24 Ci mmol^−1^; 777 to 888 GBq mmol^−1^) was purchased from PerkinElmer Life Sciences. Inositol isomers and derivatives were obtained from Sigma Aldrich (St. Louis, MO) (*allo*-, d-*chiro*-, l-*chiro*-, *epi*-, *muco*-, and *myo-*inositol, phytic acid, and *neo*-inositol, and quebrachitol), Calbiochem (*scyllo*- and *myo-*inositol), or Industrial Research Ltd. (Lower Hutt, New Zealand) (l-*chiro*-inositol, d-ononitol, d-pinitol, and viburnitol). Monosaccharides, ionophores, and other inhibitors were purchased from Sigma Aldrich and were of the highest grade available.

### Inositol uptake assays.

For uptake assays, overnight cultures of *P. carinii* (as described above) (5 × 10 ^7^ to 1 × 10^8^ cells ml^−1^) were harvested by centrifugation at 2,000 × *g* for 10 min, washed twice in phosphate-buffered saline (PBS; 135 mM NaCl, 1.3 mM KCl, 3.2 mM Na_2_HPO_4_, 0.5 mM KH_2_PO_4_, pH 7.4) at 4°C, and resuspended in PBS, unless otherwise stated. After 10 min of preincubation at 37°C, the uptake of radiolabeled *myo-*inositol was initiated by the addition of 100 μl of cell suspension (5 × 10^7^) to 100 μl of *myo*-[2-^3^H]inositol (3 μCi ml^−1^; 110 kBq ml^−1^) at 0.5 μM final concentration in PBS at 37°C. At various time points (1, 3, 5, 7, and 10 min), uptake was terminated by the addition of ice-cold PBS, followed by centrifugation at 2,000 × *g*. The pelleted organisms were snap frozen and then lysed with 0.1 M NaOH and added to 3 ml scintillation fluid (Scintiverse BD; Fisher Scientific). Radioactivity was measured by scintillation counting using a Packard TriCarb 2100 TR liquid scintillation analyzer (PerkinElmer Life Sciences). The uptake of radiolabeled *myo-*inositol at each time point was calculated and plotted as a function of time. The initial *myo-*inositol uptake rate was determined by linear regression analysis of the plotted data points for each assay within the first 10 min of *myo-*inositol uptake. Determination of picomoles per 5 × 10^7^ *P. carinii* cells was based on a standard curve of varying concentrations of *myo-*inositol and the resultant counts per minute (cpm) (*R*^2^ = 0.9989).

### Substrate saturation kinetics.

Kinetic studies for *myo-*inositol transport in *P. carinii* were performed in PBS, pH 7.4, within the linear uptake range of each concentration of *myo-*inositol, which ranged from 50 µM to 3 mM. Six biological replicates were used, based on an initial power analysis. The apparent *K*_*m*_ and *V*_max_ values were determined by the Michaelis-Menten equation *V* = *V*_max_[*S*]/(*K*_*m*_ + [*S*]), where *S* is the *myo-*inositol concentration. The data were background corrected and fitted using a 4-parameter regression in Kaleidagraph (Synergy Software, Reading, PA). The regression coefficient for the Michaelis-Menten equation fit was 0.9677.

### Ion specificity and pharmacology studies.

The ion specificity of *myo-*inositol transport in *P. carinii* was determined by first washing 5 × 10^7^ *P. carinii* cells in each of the uptake buffers of 140 mM NaCl, KCl, or choline chloride in 25 mM HEPES, pH 7.3, prior to the addition of the radiolabeled *myo-*inositol as described above. For assessment of the effect of pH (H^+^ concentration), the *P. carinii* cells were washed in each of three pH buffers representing differing proton concentrations, 316 nM H^+^ (pH 6.5), 31 nM H^+^ (pH 7.5), and 3.2 nM H^+^ (pH 8.5).

For the inhibitor (pharmacological profile) studies, the drugs carbonilcyanide, dinitrophenol, sodium azide, cytochalasin B, and phloridzin were incubated with 5 × 10^7^ ml^−1^ *P. carinii* at their final concentrations (noted in Fig. 3B) for 10 min at 37°C prior to the start of the uptake assays.

### Substrate specificity.

The substrate specificities of *P. carinii myo-*inositol and glucose transport were examined by testing 8 stereoisomers of *myo-*inositol, 5 inositol derivatives, and 15 sugars for their ability to compete with *myo-*inositol and glucose uptake. Cryopreserved *P. carinii* organisms were prepared as described above. Radiolabeled *myo-*inositol or radiolabeled glucose was added as described above, followed by incubation at 37°C for 10 min. The final concentrations of isotopes were 5 μM d-[^14^C]glucose and 0.5 μM [^3^H]*myo-*inositol. To stop uptake, iced PBS was added. Samples were washed twice in cold PBS and centrifuged at 2,000 × *g* at 4°C. Pellets were suspended in 100 µl 0.1 M NaOH, and the lysate was transferred to 3 ml scintillation fluid. The cpm were determined with a Beckman LS6500 liquid scintillation counter.

### *In vitro* ATP assay.

Lithium chloride, amitriptyline, carbamazepine, valproate, and l-fucose were evaluated for their ability to reduce the viability of *P. carinii* using the ATP bioluminescence assay established in our laboratory (28). All were also evaluated for their ability to inhibit the uptake of *myo*-[2-^3^H]inositol, as described above. The ATP assay was initiated by rapidly thawing cryopreserved *P. carinii* organisms at 37°C and resuspending them at 5 × 10^7^ nuclei/ml in RPMI 1640 containing 10% horse serum with or without a test drug. Each drug concentration was assayed in triplicate in 12-well plates. Negative controls included medium with *P. carinii* and without drug, medium with *P. carinii* and 10 μg of ampicillin/ml (negative control), and medium with *P. carinii* without drug and with any vehicles used. Pentamidine at 1 μg/ml served as the positive control for drug activity. The plates were incubated at 5% CO_2_, 37°C, and ATP content was measured at 24, 48, and 72 h postinoculation.

ATP was measured using a bioluminescence assay as previously described (48). The linear range of the ATP assay is 1 μM to 100 fM (~20,000,000 to 2,000 RLU). Fifty-microliter samples were lysed, transferred to opaque white plates, and assessed for ATP content using ATPlite-M for light emission at 562 nm, measured with a PolarStar Optima spectrophotometer (BMG, Inc.). A quench control to evaluate effects on the enzyme-substrate reaction was run for every drug tested.

### Protein structure prediction and annotation.

Protein structure modeling and annotation are based on the *P. carinii* ITR1 sequence (RefSeq accession number AIU34725.1). For comparison, ITR1 3-D structures were modeled from *Saccharomyces cerevisiae* (RefSeq accession number NP_010785.1; ScITR1), *Schizosaccharomyces pombe* (RefSeq accession number NP_593858.2; SpITR1), *Candida tropicalis* (RefSeq accession number XP_002547329.1; CtITR1), and *Fusarium oxysporum* (GenBank accession number ENH74095.1; FoITR1). 3-D structures were modeled using the Phyre2 server (49). The advantage of the server is that its search for templates includes both sequence homology and similarity in the secondary structure profile, enabling the identification of similar folds even without high sequence identity. Prediction of the transmembrane regions and membrane topology was conducted using HMMTop (50), TMPred (http://www.ch.embnet.org/software/TMPRED_form.html), MINNOU (51), CCTOP (52), and Phyre2 (49). Protein structure classification was performed using the NCBI Conserved Domain Database (33).

## SUPPLEMENTAL MATERIAL

Figure S1  3-D models of ITR1 from selected organisms. All models were generated using Phyre2. Structure alignment and rendering were performed using PyMol. Colors are used to represent source organisms as follows: green, *P. carinii* (PcITR); red, *S. cerevisiae* (ScITR1); blue, *S. pombe* (SpITR1); magenta, *C. tropicalis* (CtITR1); cyan, *F. oxysporum* (FoITR1). PcITR1 has sequence identities of 27%, 26%, 25%, and 25% to FoITR1, CtITR1, ScITR1, and SpITR1, respectively. Nevertheless, Phyre2 found that they all share the same 3-D fold with 12 membrane-spanning regions. Distances in the 3-D alignment of transmembrane regions of other ITR1s to the PcITR1 model range within RMSD values of 2.6 to 3.3 Å. Download Figure S1, PDF file, 0.03 MB

Figure S2  Prediction of the membrane topology of PcITR1. Methods used were Phyre2 (based on a 3-D structure), HMMTop, TMPred, and MINNOU (sequence based), and CCTOP (consensus based). Abbreviation of states are as follows: H, membrane α-helix; -, nonmembrane region; o, non-membrane extracellular region; i (or I), nonmembrane cytosolic region. Download Figure S2, PDF file, 0.1 MB
