# Supplementary figures and images for: Functional Characterization of Pneumocystis carinii Inositol Transporter 1
Source: mBio. 2016 Dec 13;7(6):e01851-16. doi: 10.1128/mBio.01851-16 (PMC5156303; doi:10.1128/mBio.01851-16)

Figure 1S.

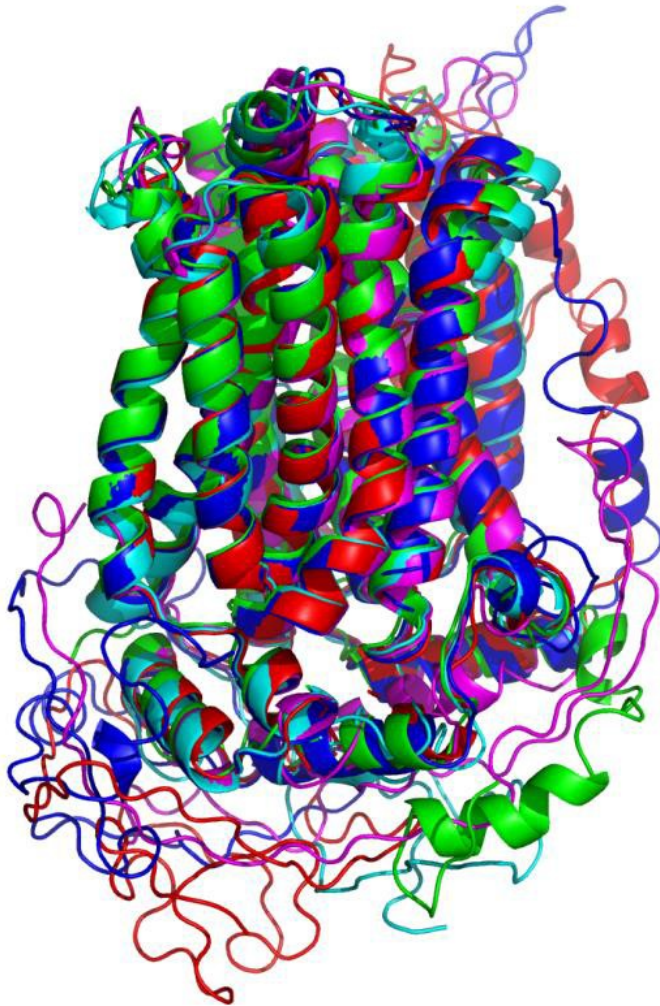

Supplement: Figure S1 — 3-D models of ITR1 from selected organisms. All models were generated using Phyre2. Structure alignment and rendering were performed using PyMol. Colors are used to represent source organisms as follows: green, P. carinii (PcITR); red, S. cerevisiae (ScITR1); blue, S. pombe (SpITR1); magenta, C. tropicalis (CtITR1); cyan, F. oxysporum (FoITR1). PcITR1 has sequence identities of 27%, 26%, 25%, and 25% to FoITR1, CtITR1, ScITR1, and SpITR1, respectively. Nevertheless, Phyre2 found that they all share the same 3-D fold with 12 membrane-spanning regions. Distances in the 3-D alignment of transmembrane regions of other ITR1s to the PcITR1 model range within RMSD values of 2.6 to 3.3 Å. Download [file mbo006163109sf1.pdf]
