# Supplementary material for: Functional Characterization of Pneumocystis carinii Inositol Transporter 1
Source: mBio. 2016 Dec 13;7(6):e01851-16. doi: 10.1128/mBio.01851-16 (PMC5156303; doi:10.1128/mBio.01851-16)
Supplement: Figure S2 — Prediction of the membrane topology of PcITR1. Methods used were Phyre2 (based on a 3-D structure), HMMTop, TMPred, and MINNOU (sequence based), and CCTOP (consensus based). Abbreviation of states are as follows: H, membrane α-helix; -, nonmembrane region; o, non-membrane extracellular region; i (or I), nonmembrane cytosolic region. Download [file mbo006163109sf2.pdf]

Figure 2S.

Annotation tool **Sequence and annotation**

```
        MYTIYHPVAIAAAATTGAMLFGFDISSMSAFVTAPQYLEYFNRPSSLLQGVITSSMPIGS 60
Phyre2  -----HHHHHHHHHHHHHHHHHHHHHHHH-------------------HHHHHHHHHHHH
HMMTop  OooooooHHHHHHHHHHHHHHHHHHHHHHHHHiiiiiiiiiiiiHHHHHHHHHHHHHHHH
TMPred  iiHHHHHHHHHHHHHHHHHHHHHoooooooooooooooooooooooooooHHHHHHHHHH
MINNOU  ---HHHHHHHHHHHHHHHHHHHHHHHHHHHHHHH------------HHHHHHHHHHHHHH
CCTOP   iiiiHHHHHHHHHHHHHHHHHHHHHHoooooooooooooooooooooHHHHHHHHHHHHH

        AIASFFSGYVTNKLGHRSINQIGAWIWIVGSIVQATSINAVHLIIGRFISSIAIGLCSSQ 120
Phyre2  HHHHHHHHH--------HHHHHHHHHHHHHHHHHHH------HHHHHHHHHHHHHHHHHH
HMMTop  HHHHHHHooooooooooooooHHHHHHHHHHHHHHHHHHHHHHHHHiiiiiiiiiiiiii
TMPred  HHHHHHHHHHHHiiiiiHHHHHHHHHHHHHHHHHHHHHooooHHHHHHHHHHHHHHHHHH
MINNOU  HHHHHHHHHHHHH---HHHHHHHHHHHHHHHHHHH---HHHHHHHHHHHHHHHHHHHHHH
CCTOP   HHHHHHHiiiiiiiiiiiiHHHHHHHHHHHHHHHHoooooooHHHHHHHHHHHHHHHHHH

        IPVWISELSPKKYRGSLVSIFQWSITWGICIMFYLSYGCTKIKGPLSFRVAWGLQVVPGF 180
Phyre2  HH---------------HHHHHHHHHHHHHHHHHHHHH----------HHHHHHHHHHHH
HMMTop  iIIIIIIIIiiiiiiHHHHHHHHHHHHHHHHHHHHooooooooooooooHHHHHHHHHHH
TMPred  HHHHHiiiiiiiiiHHHHHHHHHHHHHHHHHHHHHooooooooooooooooHHHHHHHHH
MINNOU  HHHHHHH------HHHHHHHHHHHHHHHHHHHHHHHHHH-------HHHHHHHHHHHHHH
CCTOP   HiiiiiiiiiiiiiiiiHHHHHHHHHHHHHHHHHHHHHHooooooooooHHHHHHHHHHH

        IFSFLVLFLPQSPRWLASHDRWEEAQFIISKVNNSNNFNSPEVVSEIKDIKEAVESDKDS 240
Phyre2  HHHHHHHHHHH-------------------------------------------------
HMMTop  HHHHHHHHHiiiiiiiiiiiiiiiIIIIIIIIIIIIIIIIIIIIIIIIIIIIIIIIIIII
TMPred  HHHHHHHHHiiiiiiiiiiiiiiiiiiiiiiiiiiiiiiiiiiiiiiiiiiiiiiiiiii
MINNOU  HHHHHHHH----------------------------------------------------
CCTOP   HHHHHHHiiiiiiiiiiiiiiiiiiiiiiiiiiiiiiiiiiiiiiiiiiiiiiiiiiiii

        VGYLALFSKGSRTRTFVGFFTQVWQQFTGMNIIMYYVTNVFQMAGYSGVSNLLISSIQYV 300
Phyre2  -----------HHHHHHHHHHHHHHHHHHHHHHHHHH-------------HHHHHHHHHH
HMMTop  iiiiiiiiiiiiiiiHHHHHHHHHHHHHHHHHHHooooooooooooooHHHHHHHHHHHH
TMPred  iiiiiiiiiiiiiiiiHHHHHHHHHHHHHHHHHHHHHooooooooooooooHHHHHHHHH
MINNOU  -----------HHHHHHHHHHHHHHHHHHHHHHHHHHHHHHHH------HHHHHHHHHHH
CCTOP   iiiiiiiiiiiiiiiiHHHHHHHHHHHHHHHHHHHHHHooooooooooooooHHHHHHHH

        LNVIMTVPALLFIDRWGRRPTFIYGAVLMAILLFAESIFMAIGGHYVPSYHGSEYIRWTM 360
Phyre2  HHHHHHHHHHHH------HHHHHHHHHHHHHHHHHHHHHH--------------------
HMMTop  HHHHHHHHHHHHHiiiiiiiiHHHHHHHHHHHHHHHHHHHHHHooooooooooooooooo
TMPred  HHHHHHHHHHHHHHiiiiiHHHHHHHHHHHHHHHHHHHHHHHHooooooooooooooooo
MINNOU  HHHHHHHHHHHHHHH-----HHHHHHHHHHHHHHHHHHHHHH------------------
CCTOP   HHHHHHHHHHHHHiiiiiiiHHHHHHHHHHHHHHHHHHHHHHHHHHHHoooooooooooo

        EGHRNLANAVIACSYLFVCVFAPTWGPCAWIYVSEIFPHNQRTKANGFCTFAYWTFNFIL 420
Phyre2  -------HHHHHHHHHHHHHHHHHHHHHHHHHHHHH--------HHHHHHHHHHHHHHHH
HMMTop  oooooooooHHHHHHHHHHHHHHHHHHHHHHHHHiiiiiiiiiiiiHHHHHHHHHHHHHH
TMPred  oooooHHHHHHHHHHHHHHHHHHHHHHHHHHHHiiiiiiiiiiiiiHHHHHHHHHHHHHH
MINNOU  ----HHHHHHHHHHHHHHHHHHHHHHHHHH-----------HHHHHHHHHHHHHHHHHHH
CCTOP   oooooooooHHHHHHHHHHHHHHHHHHHHHHiiiiiiiiiiiiiiiiHHHHHHHHHHHHH

        AIFVPTAFKNIQWKTFLIFATFSVTMAIHVYLMFPETCGRSLEEIEQIWRDKVPAWKTKV 480
Phyre2  HHHHHHHHH----HHHHHHHHHHHHHHHHHHHHH--------------------------
HMMTop  HHHHHHoooooooooHHHHHHHHHHHHHHHHHHHHiiiiiiiiiiiiiiiIIIIIIIIII
TMPred  HHHHHHHHooHHHHHHHHHHHHHHHHHHHHHiiiiiiiiiiiiiiiiiiiiiiiiiiiii
MINNOU  HHHHHHHHH-----HHHHHHHHHHHHHHHHHH----------------------------
CCTOP   HHHHHHHHHoooooHHHHHHHHHHHHHHHHHHiiiiiiiiiiiiiiiiiiiiiiiiiiii

        YRPDKQKSLEKKEESLLDNENENKYQV 507
Phyre2  ---------------------------
HMMTop  IIIIIIIIIIIIIIIIIIIIIIIIIII
TMPred  iiiiiiiiiiiiiiiiiiiiiiiiiii
MINNOU  ---------------------------
CCTOP   iiiiiiiiiiiiiiiiiiiiiiiiiii
```
